# 2-Amino-5-oxo-4-phenyl-5,6,7,8-tetra­hydro-4*H*-chromene-3-carbonitrile

**DOI:** 10.1107/S1600536811008130

**Published:** 2011-03-09

**Authors:** Xiaoli Wang

**Affiliations:** a School of Petrochemical Engineering, Shenyang University of Technology, Liaoyang 111003, People’s Republic of China

## Abstract

In the title mol­ecule, C_16_H_14_N_2_O_2_, the fused cyclo­hexene and pyran rings adopt an envelope and a flattened boat conformation, respectively. In the crystal, N—H⋯N and N—H⋯O hydrogen bonds link the mol­ecules into corrugated sheets parallel to the *bc* plane.

## Related literature

For the biological activities of substituted pyran derivatives, see: Lokaj *et al.* (1990 ▶); Marco *et al.* (1993 ▶). For the crystal structure of a related compound, see: Tu *et al.* (2001 ▶).
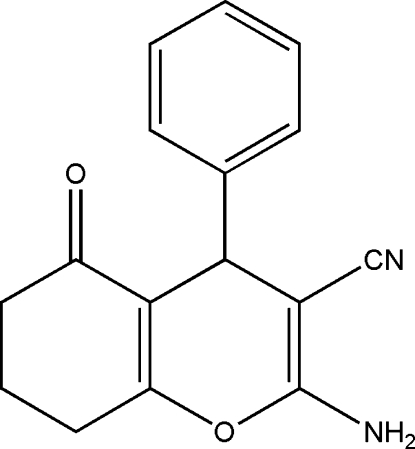


## Experimental

### 

#### Crystal data

C_16_H_14_N_2_O_2_
*M* *_r_* = 266.29
Monoclinic, 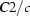

*a* = 20.210 (2) Å
*b* = 8.8161 (5) Å
*c* = 16.3862 (13) Å
β = 99.537 (1)°
*V* = 2879.2 (4) Å^3^
*Z* = 8
Mo *K*α radiation
μ = 0.08 mm^−1^
*T* = 298 K
0.32 × 0.21 × 0.15 mm

#### Data collection

Bruker SMART APEX CCD area-detector diffractometer
Absorption correction: multi-scan (*SADABS*; Sheldrick, 1996 ▶) *T*_min_ = 0.974, *T*_max_ = 0.988
7077 measured reflections
2535 independent reflections
1083 reflections with *I* > 2σ(*I*)
*R*_int_ = 0.063

#### Refinement

*R*[*F* ^2^ > 2σ(*F* ^2^)] = 0.045
*wR*(*F* ^2^) = 0.114
*S* = 0.81
2535 reflections
182 parameters
H-atom parameters constrained
Δρ_max_ = 0.12 e Å^−3^
Δρ_min_ = −0.11 e Å^−3^

### 

Data collection: *SMART* (Bruker, 2007 ▶); cell refinement: *SAINT* (Bruker, 2007 ▶); data reduction: *SAINT*; program(s) used to solve structure: *SHELXS97* (Sheldrick, 2008 ▶); program(s) used to refine structure: *SHELXL97* (Sheldrick, 2008 ▶); molecular graphics: *XP* in *SHELXTL* (Sheldrick, 2008 ▶); software used to prepare material for publication: *SHELXL97*.

## Supplementary Material

Crystal structure: contains datablocks I, global. DOI: 10.1107/S1600536811008130/cv5040sup1.cif

Structure factors: contains datablocks I. DOI: 10.1107/S1600536811008130/cv5040Isup2.hkl

Additional supplementary materials:  crystallographic information; 3D view; checkCIF report

## Figures and Tables

**Table 1:** Hydrogen-bond geometry (Å, °)

*D*—H⋯*A*	*D*—H	H⋯*A*	*D*⋯*A*	*D*—H⋯*A*
N1—H1*A*⋯N2^i^	0.86	2.16	3.007 (3)	170
N1—H1*B*⋯O2^ii^	0.86	2.00	2.848 (2)	169

Symmetry codes: (i) 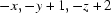; (ii) 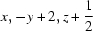.

**Fig. 1.**
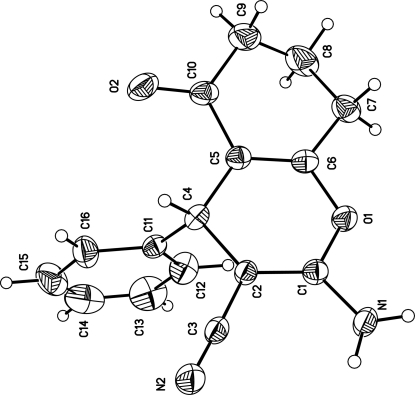
View of (I) showing the atomic numbering and 30% probability displacement ellipsoids.

## supplementary crystallographic information

### Comment

Much interest has recently been paid to the design of polyfunctionalized substituted pyran derivatives, owing to their wide range of biological activities (Lokaj *et al.*, 1990; Marco *et al.*, 1993). We obtained the title compound, (I), and report here its crystal structure.

In (I) (Fig. 1), the bond lengths and angles of the main molecule are normal and correspond to those observed in 2-amino-7,7-dimethyl- 5-oxo-4-phenyl-5,6,7,8-tetra- hydro-4*H*-chromene-3-carbonitrile (Tu *et al.*, 2001). The fused cyclohexene and pyran rings adopt an envelope and a flattened bath conformations, respecteviley. The dihedral angle between the O1/C1/C2/C5/C6 and C2/C4/C5 planes is 16.67 (14) °. The O1/ C1/C2/C5/C6 plane forms an angle of 89.01 (8)° with the phenyl plane. In the crystal, the nitrile group is typical [N≡C = 1.146 (3) Å] and the carbonyl group also is reasonable [C═O =1.228 (3) Å]. The C5/C6/C7/C8/C9/C10 plane also adopt an chair configuration in the compound, and the the dihedral angle between the C5/C6/C7/C9/C10 plane and the C7/C8/C9 plane is 46.14 (3)°.

In the crystal structure, there exist typical intermolecular N—H···O and N—H···N hydrogen bonds (Table 1). The amino N1 atom of one molecule links through H1B to the nitrile N2 atom of another molecule, creating a dimer. The amino N1 atom of one molecule also links through H1A to the keto O2 atom of another molecule to form the two-dimensional framework.

### Experimental

Malononitrile (10 mmol), 1,3-cyclohexanedione (10 mmol),and benzaldehyde(10 mmol)was dissolved in 20 ml e thanol ml in a round-bottom flask. The mixture was warmed, with agitation, to 353 K over a period of 3 h. The resulting solution was cooled. Crystal of (I) suitable for X-ray diffraction analysis were obtained by slow evaporation.

### Refinement

All H atoms were placed in geometrically idealized positions (N—H 0.86 and C—H 0.93–0.98 Å) and treated as riding on their parent atoms, with *U*_iso_(H) = 1.2*U*_eq_(C) (C,*N*).

### Crystal data

**Table:**

C_16_H_14_N_2_O_2_	*F*(000) = 1120
*M**_r_* = 266.29	*D*_x_ = 1.229 Mg m^−^^3^
Monoclinic, *C*2/*c*	Mo *K*α radiation, λ = 0.71073 Å
*a* = 20.210 (2) Å	Cell parameters from 851 reflections
*b* = 8.8161 (5) Å	θ = 2.5–19.1°
*c* = 16.3862 (13) Å	µ = 0.08 mm^−^^1^
β = 99.537 (1)°	*T* = 298 K
*V* = 2879.2 (4) Å^3^	Block, red
*Z* = 8	0.32 × 0.21 × 0.15 mm

### Data collection

**Table:**

Bruker SMART APEX CCD area-detector diffractometer	2535 independent reflections
Radiation source: fine-focus sealed tube	1083 reflections with *I* > 2σ(*I*)
graphite	*R*_int_ = 0.063
φ and ω scans	θ_max_ = 25.0°, θ_min_ = 2.0°
Absorption correction: multi-scan (*SADABS*; Sheldrick, 1996)	*h* = −24→17
*T*_min_ = 0.974, *T*_max_ = 0.988	*k* = −10→10
7077 measured reflections	*l* = −19→19

### Refinement

**Table:**

Refinement on *F*^2^	Secondary atom site location: difference Fourier map
Least-squares matrix: full	Hydrogen site location: inferred from neighbouring sites
*R*[*F*^2^ > 2σ(*F*^2^)] = 0.045	H-atom parameters constrained
*wR*(*F*^2^) = 0.114	*w* = 1/[σ^2^(*F*_o_^2^) + (0.0454*P*)^2^] where *P* = (*F*_o_^2^ + 2*F*_c_^2^)/3
*S* = 0.81	(Δ/σ)_max_ = 0.001
2535 reflections	Δρ_max_ = 0.12 e Å^−^^3^
182 parameters	Δρ_min_ = −0.11 e Å^−^^3^
0 restraints	Extinction correction: *SHELXL97* (Sheldrick, 2008), Fc^*^=kFc[1+0.001xFc^2^λ^3^/sin(2θ)]^-1/4^
Primary atom site location: structure-invariant direct methods	Extinction coefficient: 0.0017 (2)

### Special details

**Table:**

Geometry. All e.s.d.'s (except the e.s.d. in the dihedral angle between two l.s. planes) are estimated using the full covariance matrix. The cell e.s.d.'s are taken into account individually in the estimation of e.s.d.'s in distances, angles and torsion angles; correlations between e.s.d.'s in cell parameters are only used when they are defined by crystal symmetry. An approximate (isotropic) treatment of cell e.s.d.'s is used for estimating e.s.d.'s involving l.s. planes.
Refinement. Refinement of *F*^2^ against ALL reflections. The weighted *R*-factor *wR* and goodness of fit *S* are based on *F*^2^, conventional *R*-factors *R* are based on *F*, with *F* set to zero for negative *F*^2^. The threshold expression of *F*^2^ > σ(*F*^2^) is used only for calculating *R*-factors(gt) *etc*. and is not relevant to the choice of reflections for refinement. *R*-factors based on *F*^2^ are statistically about twice as large as those based on *F*, and *R*- factors based on ALL data will be even larger.

### Fractional atomic coordinates and isotropic or equivalent isotropic displacement parameters (Å^2^)

**Table:**

	*x*	*y*	*z*	*U*_iso_*/*U*_eq_	
O1	0.10075 (8)	0.98207 (19)	1.01594 (9)	0.0666 (5)	
C1	0.07153 (12)	0.8416 (3)	1.00139 (16)	0.0571 (7)	
C5	0.09662 (11)	1.0170 (3)	0.87111 (15)	0.0550 (7)	
C2	0.05849 (11)	0.7806 (3)	0.92527 (13)	0.0501 (6)	
N1	0.05983 (10)	0.7810 (2)	1.07271 (11)	0.0759 (7)	
H1A	0.0419	0.6926	1.0729	0.091*	
H1B	0.0703	0.8305	1.1182	0.091*	
C4	0.08060 (11)	0.8555 (3)	0.85109 (13)	0.0549 (7)	
H4	0.0427	0.8528	0.8053	0.066*	
O2	0.08103 (10)	1.0779 (2)	0.73126 (12)	0.0897 (7)	
C10	0.09649 (13)	1.1227 (3)	0.80287 (19)	0.0686 (8)	
C11	0.13891 (14)	0.7707 (3)	0.82384 (15)	0.0569 (7)	
C3	0.02554 (13)	0.6406 (4)	0.91435 (14)	0.0586 (7)	
C6	0.10774 (12)	1.0700 (3)	0.94811 (17)	0.0604 (7)	
N2	−0.00226 (12)	0.5272 (3)	0.90338 (13)	0.0839 (8)	
C7	0.12867 (14)	1.2263 (3)	0.97399 (16)	0.0790 (8)	
H7A	0.1622	1.2225	1.0237	0.095*	
H7B	0.0903	1.2825	0.9865	0.095*	
C9	0.11266 (18)	1.2848 (4)	0.82299 (19)	0.1061 (11)	
H9A	0.0712	1.3400	0.8232	0.127*	
H9B	0.1346	1.3279	0.7799	0.127*	
C16	0.13116 (16)	0.6985 (3)	0.74839 (18)	0.0867 (9)	
H16	0.0904	0.7056	0.7127	0.104*	
C8	0.15751 (17)	1.3064 (3)	0.9057 (2)	0.1100 (12)	
H8A	0.1621	1.4139	0.9181	0.132*	
H8B	0.2018	1.2663	0.9029	0.132*	
C12	0.19982 (16)	0.7600 (3)	0.87459 (18)	0.0894 (10)	
H12	0.2064	0.8083	0.9258	0.107*	
C15	0.1828 (2)	0.6155 (4)	0.7246 (3)	0.1201 (14)	
H15	0.1766	0.5667	0.6736	0.144*	
C14	0.2420 (2)	0.6057 (5)	0.7757 (3)	0.1254 (17)	
H14	0.2767	0.5492	0.7600	0.150*	
C13	0.25165 (18)	0.6783 (5)	0.8505 (3)	0.1215 (14)	
H13	0.2930	0.6727	0.8850	0.146*	

### Atomic displacement parameters (Å^2^)

**Table:**

	*U*^11^	*U*^22^	*U*^33^	*U*^12^	*U*^13^	*U*^23^
O1	0.0811 (13)	0.0612 (12)	0.0571 (11)	−0.0126 (10)	0.0103 (9)	0.0013 (10)
C1	0.0571 (17)	0.0574 (18)	0.0580 (17)	−0.0072 (14)	0.0135 (14)	0.0012 (15)
C5	0.0524 (16)	0.0553 (18)	0.0593 (17)	0.0015 (13)	0.0151 (13)	0.0061 (15)
C2	0.0532 (16)	0.0537 (17)	0.0443 (15)	−0.0037 (13)	0.0104 (12)	−0.0004 (13)
N1	0.1060 (19)	0.0758 (16)	0.0488 (13)	−0.0279 (13)	0.0216 (13)	−0.0051 (12)
C4	0.0530 (16)	0.0653 (18)	0.0462 (15)	−0.0032 (14)	0.0073 (12)	0.0070 (13)
O2	0.1089 (16)	0.0947 (16)	0.0703 (13)	0.0135 (12)	0.0287 (13)	0.0276 (12)
C10	0.071 (2)	0.063 (2)	0.078 (2)	0.0115 (15)	0.0308 (18)	0.0157 (18)
C11	0.0616 (18)	0.0584 (17)	0.0533 (16)	−0.0055 (14)	0.0177 (15)	0.0073 (14)
C3	0.0683 (18)	0.066 (2)	0.0439 (16)	−0.0033 (16)	0.0159 (14)	0.0034 (14)
C6	0.0599 (18)	0.0542 (18)	0.0675 (18)	−0.0019 (14)	0.0115 (14)	0.0077 (16)
N2	0.110 (2)	0.0755 (18)	0.0680 (16)	−0.0249 (16)	0.0210 (14)	−0.0018 (14)
C7	0.084 (2)	0.062 (2)	0.093 (2)	−0.0100 (16)	0.0226 (17)	−0.0057 (17)
C9	0.150 (3)	0.072 (2)	0.110 (3)	0.007 (2)	0.061 (2)	0.019 (2)
C16	0.100 (3)	0.088 (2)	0.076 (2)	−0.0078 (18)	0.0265 (18)	−0.0177 (18)
C8	0.137 (3)	0.064 (2)	0.141 (3)	−0.031 (2)	0.058 (3)	−0.008 (2)
C12	0.066 (2)	0.116 (3)	0.088 (2)	0.015 (2)	0.018 (2)	0.0002 (19)
C15	0.162 (4)	0.091 (3)	0.130 (4)	0.003 (3)	0.089 (3)	−0.020 (2)
C14	0.119 (4)	0.101 (3)	0.181 (5)	0.021 (3)	0.098 (4)	0.025 (3)
C13	0.071 (2)	0.144 (4)	0.155 (4)	0.023 (2)	0.036 (3)	0.018 (3)

### Geometric parameters (Å, °)

**Table:**

O1—C1	1.376 (3)	C6—C7	1.482 (3)
O1—C6	1.381 (3)	C7—C8	1.520 (3)
C1—N1	1.341 (3)	C7—H7A	0.9700
C1—C2	1.344 (3)	C7—H7B	0.9700
C5—C6	1.329 (3)	C9—C8	1.512 (4)
C5—C10	1.455 (3)	C9—H9A	0.9700
C5—C4	1.484 (3)	C9—H9B	0.9700
C2—C3	1.400 (3)	C16—C15	1.382 (4)
C2—C4	1.514 (3)	C16—H16	0.9300
N1—H1A	0.8600	C8—H8A	0.9700
N1—H1B	0.8600	C8—H8B	0.9700
C4—C11	1.524 (3)	C12—C13	1.382 (4)
C4—H4	0.9800	C12—H12	0.9300
O2—C10	1.228 (3)	C15—C14	1.344 (5)
C10—C9	1.491 (4)	C15—H15	0.9300
C11—C12	1.370 (3)	C14—C13	1.368 (5)
C11—C16	1.376 (3)	C14—H14	0.9300
C3—N2	1.146 (3)	C13—H13	0.9300
			
C1—O1—C6	117.64 (19)	C8—C7—H7A	109.6
N1—C1—C2	127.8 (2)	C6—C7—H7B	109.6
N1—C1—O1	109.9 (2)	C8—C7—H7B	109.6
C2—C1—O1	122.3 (2)	H7A—C7—H7B	108.1
C6—C5—C10	118.9 (3)	C10—C9—C8	113.3 (3)
C6—C5—C4	122.9 (2)	C10—C9—H9A	108.9
C10—C5—C4	118.1 (2)	C8—C9—H9A	108.9
C1—C2—C3	119.2 (2)	C10—C9—H9B	108.9
C1—C2—C4	122.1 (2)	C8—C9—H9B	108.9
C3—C2—C4	118.6 (2)	H9A—C9—H9B	107.7
C1—N1—H1A	120.0	C11—C16—C15	121.3 (3)
C1—N1—H1B	120.0	C11—C16—H16	119.4
H1A—N1—H1B	120.0	C15—C16—H16	119.4
C5—C4—C2	108.9 (2)	C9—C8—C7	110.8 (3)
C5—C4—C11	112.61 (19)	C9—C8—H8A	109.5
C2—C4—C11	111.50 (19)	C7—C8—H8A	109.5
C5—C4—H4	107.9	C9—C8—H8B	109.5
C2—C4—H4	107.9	C7—C8—H8B	109.5
C11—C4—H4	107.9	H8A—C8—H8B	108.1
O2—C10—C5	119.7 (3)	C11—C12—C13	120.6 (3)
O2—C10—C9	122.1 (3)	C11—C12—H12	119.7
C5—C10—C9	118.1 (3)	C13—C12—H12	119.7
C12—C11—C16	118.1 (3)	C14—C15—C16	119.7 (4)
C12—C11—C4	121.1 (2)	C14—C15—H15	120.2
C16—C11—C4	120.8 (3)	C16—C15—H15	120.2
N2—C3—C2	178.2 (3)	C15—C14—C13	120.4 (4)
C5—C6—O1	122.9 (2)	C15—C14—H14	119.8
C5—C6—C7	126.4 (2)	C13—C14—H14	119.8
O1—C6—C7	110.7 (2)	C14—C13—C12	119.9 (4)
C6—C7—C8	110.3 (2)	C14—C13—H13	120.0
C6—C7—H7A	109.6	C12—C13—H13	120.0

### Hydrogen-bond geometry (Å, °)

**Table:**

*D*—H···*A*	*D*—H	H···*A*	*D*···*A*	*D*—H···*A*
N1—H1A···N2^i^	0.86	2.16	3.007 (3)	170
N1—H1B···O2^ii^	0.86	2.00	2.848 (2)	169

Symmetry codes: (i) −*x*, −*y*+1, −*z*+2; (ii) *x*, −*y*+2, *z*+1/2.
